# Virulence of mixed fungal infections in honey bee brood

**DOI:** 10.1186/1742-9994-9-5

**Published:** 2012-03-23

**Authors:** Svjetlana Vojvodic, Jacobus J Boomsma, Jørgen Eilenberg, Annette B Jensen

**Affiliations:** 1 Center for Social Evolution, Department of Agriculture and Ecology Faculty of Life Sciences, University of Copenhagen, Thorvaldsensvej 40, DK 1871 Frederiksberg C, Denmark; 2 Current address: Center for Insect Science, University of Arizona 1007 E. Lowell Street, P.O. Box 210106, Tucson AZ 85721-0106, USA; 3 Centre for Social Evolution, Department of Biology, University of Copenhagen, Universitetsparken 15, 2100 Copenhagen, Denmark

**Keywords:** *Apis mellifera*, *Ascosphaera*, Competition, Mixed infections, Virulence

## Abstract

**Introduction:**

Honey bees, *Apis mellifera*, have a diverse community of pathogens. Previous research has mostly focused on bacterial brood diseases of high virulence, but milder diseases caused by fungal pathogens have recently attracted more attention. This interest has been triggered by partial evidence that co-infection with multiple pathogens has the potential to accelerate honey bee mortality. In the present study we tested whether co-infection with closely related fungal brood-pathogen species that are either specialists or non-specialist results in higher host mortality than infections with a single specialist. We used a specially designed laboratory assay to expose honey bee larvae to controlled infections with spores of three *Ascosphaera *species: *A. apis*, the specialist pathogen that causes chalkbrood disease in honey bees, *A. proliperda*, a specialist pathogen that causes chalkbrood disease in solitary bees, and *A. atra*, a saprophytic fungus growing typically on pollen brood-provision masses of solitary bees.

**Results:**

We show for the first time that single infection with a pollen fungus *A. atra *may induce some mortality and that co-infection with *A. atra *and *A. apis *resulted in higher mortality of honey bees compared to single infections with *A. apis*. However, similar single and mixed infections with *A. proliperda *did not increase brood mortality.

**Conclusion:**

Our results show that co-infection with a closely related fungal species can either increase or have no effect on host mortality, depending on the identity of the second species. Together with other studies suggesting that multiple interacting pathogens may be contributing to worldwide honey bee health declines, our results highlight the importance of studying effects of multiple infections, even when all interacting species are not known to be specialist pathogens.

## Introduction

Variation in virulence (i.e. disease-induced host mortality) among pathogens is shaped by evolutionary pressure emanating from the combined life histories of pathogens and hosts [1]. However, virulence is difficult to predict when interactions are not restricted to a single host and pathogen, but involve multiple infections [2,3]. Conflict between co-infecting strains can lead to different within-host dynamics, with consequences for virulence, transmission and host resistance [4,5]. Examples illustrating aspects of this interaction-complexity are *Daphia magna *infections with bacteria *Pasteuria ramosa *and microsporidium *Octosporea bayeri *[6], helminths occupying mammalian guts [7], *Plasmodium chabaudi *clones infecting mice [8], and *Metarhizium anisopliae *var. *anisopliae *and *Aspergillus flavus *fungi infecting leaf-cutting ants [9]. Increased virulence of mixed infections can be due to increased pathogen densities [10-12] or to the host immune system being less efficient in clearing up multiple infections e.g. [13]. However, in some cases, the virulence of a mixed infection merely reflects the virulence of the most virulent strain/species e.g. [5,14,15]. The outcome of parasite interactions can also result in under-exploitation of the host and a reduction in virulence, possibly as a result of cooperation between parasites when benefits are shared [16,17].

A diverse assembly of pathogens has been described for honey bees suggesting that colonies face considerable risks of reduced productivity and death due to disease [18-20]. In addition to pathogenic microbes, a large diversity of non-pathogenic bacteria, yeasts, and molds have been found in the gut of adult honey bees [21,22], in larval feces upon pupation [23], and in dead honey bee larvae [24]. To understand the epidemiology of honey bee diseases it thus appears increasingly apposite to consider the entire community of microorganisms in which single pathogens operate, an approach that is also increasingly used in issues of human health and disease [25]. The goal of the current study was to investigate part of the honey bee microbial community, focusing on two non-pathogenic fungal species and the effect they can have on the known chronic fungal disease of honey bees, chalkbrood.

*Ascosphaera *is a fungal genus that has evolved to exclusively thrive in bee-habitats. We used *Ascosphaera apis*, an obligate specialist pathogen that causes chalkbrood disease in honey bee larvae, and two other *Ascosphaera *species that are found in association with honey bees, but are usually pathogens (*A. proliperda*) or saprophytes (*A. atra*) of solitary bee species. Chalkbrood disease develops after larvae ingest fungal spores, after which hyphal growth kills the larvae and leads to new spore formation on the cuticle of the cadavers. These are either transmitted within the colony via contaminated wax [26] and worker bees, or between colonies via contaminated pollen on flowers [21], or handling by bee keepers [27]. Solitary bees have been proposed as a potential natural reservoir of *Ascosphaera *pathogens for managed honey bees [19], but the infectiveness of other *Ascosphaera *species on honey bee larvae has never been investigated. We exposed honey bee larvae to single and mixed infections of the three *Ascosphaera *species in order to investigate pathogen-specific and combined virulence. Any potential environmental effect and presence of other microbes was minimized by artificially rearing honey bee larvae under controlled laboratory conditions.

## Results

**Figure 1:**
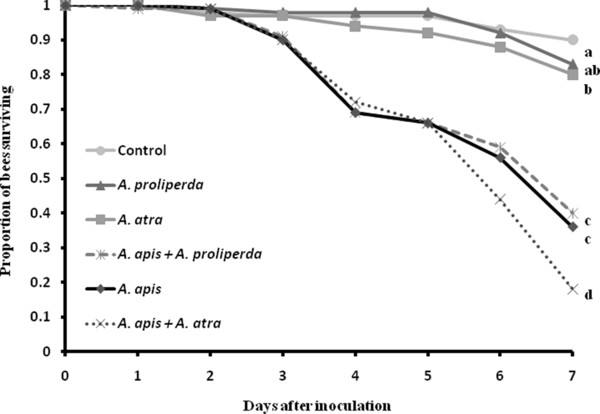
**Survival of honey bee larvae following single infections with *Ascosphaera proliperda, A. atra*, and *A. apis*, and a mixed infections with *A. apis *+ *A. proliperda *and *A. apis *+ *A. atra***. Larvae in the control were treated with distilled water and all treatments were replicated at three time periods giving a total of 540 larvae per experiment (i.e. 30 larvae × 6 treatment groups × 3 replicates). Different letters indicate significantly different survival at the last day (Day 7) of the experiment (*P <*0.05).

Mortality in the controls was less than 10% on Day 7 and no honey bee larvae in the control group died from visible *Ascosphaera *infections, validating that the experimental methodology was sound. Larvae exposed to a single species of the solitary-bee pathogen *A. proliperda *and the pollen saprophyte *A. atra *caused 17% and 20% mortality induced by either the pathogen or natural mortality, respectively. The mortality on Day 7 induced by each of the two single infections of *A. atra *and *A. proliperda *did not differ (*χ*^2 ^= 0.42, d.f. = 1, *P *= 0.519), although the analysis including the single and both of the mixed infections with *A. apis *indicated that *A. atra *did in fact cause a significantly higher mortality than the control (*χ*^2 ^= 4.24, d.f. = 1, *P *= 0.04) (Figure 1).

*Ascosphaera apis *induced a total mortality of 64% on Day 7 of the experiment, whereas *A. apis *+ *A. proliperda *did not significantly enhance mortality compared to the single infection of *A. apis (χ*^2 ^= 0.49, d.f. = 1, *P *= 0.482; the 60% mortality was in fact slightly lower). However, the *A. apis *+ *A. atra *infection treatment caused 83% mortality on the same day, significantly higher than the single *A. apis *infections (*χ*^2 ^= 9.08, d.f. = 1, *P *= 0.003), and the *A. apis *+ *A. proliperda *mixed infections (*χ*^2 ^= 13.36, d.f. = 1, *P *= 0.003).

Survival curves over a period of 7 days indicated that host survival after exposure to *A. apis *was not significantly different from survival after exposure to a mixed treatment of *A. apis *+ *A. proliperda *(Log Rank *χ*^2 ^= 0.314, d.f. = 1, *P *= 0.575). However, the survival curve after infection with *A. apis *alone differed significantly from the curve obtained from combined exposure with *A. apis *+ *A. atra *(Log Rank *χ*^2 ^= 4.480, d.f. = 1, *P *= 0.034). The two mixed treatments (*A. apis *+ *A. atra *and *A. apis *+ *A. proliperda*) were also significantly different from each other (Log Rank *χ*^2 ^= 7.126, d.f. = 1, P = 0.008).

## Discussion

*In vitro *reared honey bee larvae infected with *A. apis *predictably developed chalkbrood disease, but also infections with *A. proliperda *and *A. atra *produced disease albeit at a very low frequency. In mixed infections, combining *A. atra *and *A. apis *spores increased larval death rate significantly compared to *A. apis *treatment alone, whereas *A. proliperda *co-infection with *A. apis *had similar effect as the *A. apis *treatment alone. These differences shed interesting light on the interaction dynamics between *Ascosphaera *species and bees, as well as generate novel questions on the evolution of virulence in these fungi.

The specialized pathogen *A. apis *can be expected to have a life cycle that is exclusively adapted to infect honey bee larvae, with factors such as pH in the host gut and colony temperature and humidity affecting the details of spore germination [28,29]. The other *Ascosphaera *species used in this study have been found in association with honey bees, but they have not been previously recorded as disease-inducing pathogens of honey bee larvae [30,31], and would thus be unlikely to share any such adaptations with *A. apis*. In the current study *A. atra *strains were isolated from honey bee colonies and not from solitary bees, which demonstrates that honey bees are exposed to this solitary bee saprophyte. The three *Ascosphaera *species were selected because of their close phylogenetic relationship and their different host specialization. *Ascosphaera proliperda *spores can induce disease in the solitary leaf-cutting bee *Megachile rotundata *[32,33], but experiments on leaf-cutting bee larvae have shown that *A. atra *does not induce pathological symptoms in *Megachile *bees [34]. Furthermore, a survey of leafcutting bee larvae infected with chalkbrood has shown that ca 25% of all infected larvae were infected with the honey bee pathogen *A. apis*, in addition to *A. aggregate*, a specialist pathogen of leafcutting bees [35]. These results suggest that transmission of *Ascosphaera *pathogens among honey bees and solitary bees is likely. However, our study is the first to show that multiple *Ascosphaera *infections may also negatively affect honey bee hosts.

Mixed infections are expected to be common in nature and to act independently, synergistically, or antagonistically. These interactions are often influenced by environmental conditions and by the order in which infections happened [13,14]. The mechanisms of interactions between the specialist honey bee pathogen and the pollen saprophyte *A. atra *are unknown. We observed that a small number of larvae exposed to spores of *A. atra *and *A. proliperda *in fact had fungal mycelia growing on the larval cuticle. We were not able to determine whether this growth had caused larval death, but it was interesting to observe that several of the larvae/pupae that were covered with mycelia of *A. atra *or *A. proliperda *were still alive, suggesting that these fungi can grow superficially, possibly on the host feces. In fact the mean survival times of honey bees after infection with *A. proliperda *did not differ from control survival, contrary to the *A. atra *treatment, suggesting that the pathogenicity (pathogen ability to produce infection) of *A. proliperda *was negligible compared to *A. atra*.

Studies that investigated the virulence of mixed infections have often used virulent clones or strains of the same pathogen or closely related pathogen species. In those cases factors such as limited supply of host resources, host immune responses, or direct interference between pathogens have been the outcome of the mixed infections e.g. [9,12,13]. Few recent studies have explored the interactions among lethal and non-lethal pathogens, but those available showed that competition for resources or infection sites can occur [36] and that antagonistic competition among stains can be enhanced [37]. Even among endocommensal fungal symbionts, competition can result in the displacement of symbionts within the host, possibly due to resource competition [38]. These studies show that competitive success in mixed infections cannot easily be predicted by pathogen growth or, in the case of virulent pathogens, by host mortality rate after a single infection.

Our present study shows that significant epidemiological effects are not only caused by diseases that can wipe out entire honey bee colonies (e.g. bacterial foulbrood), but also by milder diseases such as chalkbrood that are normally not a threat to entire colonies although they are lethal to individual larvae. Recent theoretical models that explore interactions among host pathogens and non-lethal synergists have shown that host investments in disease tolerance or resistance can affect population and evolutionary dynamics [39]. Empirical studies have shown that honey bee colonies that are relatively resistant to *A. apis *have a higher concentration of symbiotic microbes (e.g. yeasts) in the pollen provision masses (bee bread) and some of these microbes (e.g. *Mucorales *molds) can apparently inhibit *A. apis *growth [21]. Furthermore, honey bee colonies vary in resistance/tolerance to chalkbrood and chalkbrood strains differ significantly in virulence [40-42]. One should keep in mind that some of these differences in resistance refer to individual larvae in the same lab-assays as in the present study e.g. [42], and not necessarily to entire colonies, as chalkbrood is generally considered to be a mild disease that can affect every colony under certain circumstances.

Future controlled laboratory assays such as the one applied in the present study may add considerable precision to our understanding of disease pressure in honey bees, not only because they allow more microorganisms to be identified as potential pathogens, but also because they may reveal co-infection synergies that could help induce more devastating stress symptoms that threaten the survival of entire colonies. The main result of our study is that co-infection with related but relatively harmless pathogens can increase host mortality beyond what specialist pathogens achieved on their own. Understanding interactions among specialist pathogens and saprophytes and the forces that drive their respective pathogenic abilities are important not only from an evolutionary perspective, but also in applied contexts, for example in the case of chalkbrood disease management.

## Conclusions

Fungal pathogens of the family *Ascosphaera *are found as pathogens of either honey bee brood or solitary bee brood, or as saprophytes on pollen. Different species of *Ascosphaera *have been found in both honey bee and solitary bee guts, but the specific and combined effects of these fungi on honey bee larvae have not been quantitatively tested. Considering the economic importance and current health threat to honey bees worldwide, knowledge of possible synergistic effects of these pathogens is warranted. Our study demonstrates that the host mortality induced by *A. apis *, causative agent of chalkbrood disease in honey bee brood, can be increased by the presence of the avirulent pollen saprophyte *A. atra*. In contrast, co-infections of the solitary bee pathogen *A. proliperda *and *A. apis *did not result in higher host mortality than single *A. apis *infections. This poses interesting questions relating to the ways in which the honey bee immune system handles (co)infections by related pathogens. Specific knowledge of the life histories and phylogenetic relationships of pathogens may therefore help to predict the outcomes of mixed infections. Insights like this are important not only for understanding host-pathogen coevolution, but also for applied perspectives in managing and protecting key pollinator species.

## Material and methods

### Maintenance of cultures and inoculum preparation

The *Ascosphaera *strains used in this study came from the USDA-ARS Collection of Entomopathogenic Fungal Cultures in Ithaca, New York, USA. Two *A. apis *strains (ARSEF 7405 and ARSEF 7406) were isolated from *A. mellifera*, whereas the *Ascosphaera proliperda *strain (ARSEF 696) was isolated from *Megachile rotundata *and the *A. atra *strain (ARSEF 5147) from honey of *A. mellifera*. The cultures of these fungal species were maintained on Sabouraud Dextrose Agar (SDA) at 25°C with monthly transfer to new plates. Of the three species of *Ascosphaera *used in this study, only *A. apis *has its sexual reproductive structures on different strains, so that strains of different mating types had to be maintained on individual plates. *Ascosphaera apis *subcultures from each of these two mating types were transferred onto a single agar plate three weeks prior to the experiment to allow sexual reproduction and spore formation. Inocula were prepared as described in [29].

Spore viability for *A. apis *was tested following the protocol of James and Buckner [43] with a few modifications. Spore suspensions were made with 2 × 10^7 ^spores per ml mixed with 150 μl GLEN, a liquid medium suitable for germination and *in vitro *growth of insect pathogenic fungi [44]. Droplets of 10 μl of this mixture were placed on each of three spot of a sterile six-well Teflon coated slide, which were then placed in a sterile Petri dish lined with wet filter paper. Each Petri dish was subsequently placed in an airtight container and flushed with CO_2 _to ensure spore activation and germination as suggested by Heath and Gaze [45]. The containers were incubated for 48 h at 34°C and the germination percentages were determined using differential interference contrast microscopy at 400 × magnification. One hundred spores were evaluated for germination by recording enlargement or germ tube formation in three different randomly chosen fields of view. The spore germination rate for *A. apis *ranged from 70 to 100%, but the exact spore germination in the host gut is hard to predict so that spore germination was used only as an indicator of the spore viability.

### Larval rearing and inoculation

Honey bee (*A. mellifera*) larvae were obtained from an apiary located at the University of Copenhagen. Colonies were checked regularly and were free of any noticeable brood or adult bee diseases. For each experiment larvae were transferred from the hive and reared *in vitro *following the protocol of Aupinel et al. [46]. Larval age was estimated by size [47] and only 1st instar larvae (which were not older then 24 h) were taken from the combs. After removal from the comb each larva was placed into an individual cell on a 48-well tissue culture plate with 20 μl of larval diet per cell. The larval diet consisted of 50% Chinese fresh frozen royal jelly (v/v) (Sonnentracht Imkerei GmbH, Bremen, Germany), 6% D-glucose (w/v), 6% D-fructose (w/v), 1% (w/v) yeast extract and sterile deionized water. The diet was mixed and frozen in smaller aliquots and was pre-heated to 34°C before being used for feeding. The larvae were fed once a day with 20 μl diet on the first day, and 40 μl on four consecutive days. The tissue culture plates with the larvae were stored in a humid bath with 80% RH and incubated at 34°C in constant darkness. Wells were gently cleaned with cotton wool when larvae defecated shortly before pupation.

Two days before the experiment larvae were removed from a colony and reared in vitro as described above. After a 48 h acclimatization period each larva was fed 5 μl of a designated spore suspension of a single pathogen, with a combination of two pathogens, or with distilled water as control. The spore dosage used was close to the LC_50 _of *A. apis*, as determined previously [29]. We doubled the stock concentrations prior to mixing pathogens so that the mixed infection of each pathogen is directly comparable to the single pathogen concentration (e.g. each single inoculum had 5 × 10^5 ^spores/ml and each mixed inoculum had 1 × 10^6 ^spores/ml with equal shares of the two pathogens). In other words the mixed spore inocula contained the same total number of spores of each pathogen as the single species inocula, but the overall concentration of spores in the mixed inocula was doubled. A total of 5 μl of spores suspended in deionized water were fed to each individual larva and the control group received deionized water without spores. Within a period of one day, the larvae ate all the diet including the spores, so that no spores remained in the wells that could have given later infection. Each treatment group (i.e. *A. apis, A. atra, A. proliperda, A. apis *+ *A. atra, A. apis *+ *A. proliperda*, and controls) was replicated at three time periods giving a total of 540 larvae per experiment (i.e. 30 larvae × 6 treatment groups × 3 replicates). The culture plates with experimental larvae were kept in a humid chamber at a constant temperature of 34°C for 7 days. Within the 7 day period (usually at day 5) honey bee larvae defecate and turn into pupae. By that time new infections are extremely unlikely as defecation expels any *Ascosphaera *spores from the gut. The number of diseased, surviving, and infected larvae were examined microscopically and recorded daily. Infected host larvae were identified by the presence of fungal hyphae on the cuticle. Larvae that died without any visual presence of fungal hyphae were re-examined the following day. If the pathogen was observed protruding through the host cuticle, these larvae were considered dead from the pathogen on the previous day. If the pathogen was not visually present on dead larvae, they were recorded as dead from natural causes.

### Statistical analysis

The effect of each fungal species on the survival of honey bee larvae was analyzed with Kaplan-Meier survivorship analysis (PROC LIFETEST, SAS ver. 9.1). Post-hoc analysis was performed on larval survival on the last day of the experiment (day 7) using a generalized linear model (PROC GENMOD, SAS ver. 9.1).

## Competing interests

The authors declare that they have no competing interests.

## Authors' contributions

SV designed experiment, performed analysis, and wrote the manuscript. JJB, JE, and ABJ contributed to the manuscript preparation. All authors read and approved the final manuscript.
